# Corticosteroid insensitivity in severe asthma: impaired nuclear translocation of glucocorticoid receptor in airway smooth muscle cells

**DOI:** 10.1186/2045-7022-3-S1-O6

**Published:** 2013-05-03

**Authors:** Pankaj Bhavsar, Pojui Chang, Charis Michaeloudes, Fan Chung

**Affiliations:** 1 Experimental Studies, National Heart & Lung Institute, Imperial College, London SW3, UK

## Background

Patients with severe asthma are less responsive to the beneficial effects of corticosteroid (CS) therapy. CS mediate their therapeutic effects through activation of the glucocorticoid receptor (GR) and suppression of NF-κB activity.

## Objectives

We investigated whether i) corticosteroid insensitivity was present in airway smooth muscle cells (ASMC) of patients with severe asthma and ii) there was a defect in the molecular action of the glucocorticoid receptor (GR) in ASMC of patients with severe asthma.

## Methods

ASMC of the healthy (12), non-severe (NSA; 10) and severe asthma (SA; 10) obtained from endobronchial biopsies were pretreated with dexamethasone (Dex) at 10^-10^-10^-6^ M followed by stimulation with TNF-α and IFN-γ (10 ng/ml each), alone or in combination (T+I). CXCL8 and CCL11 release was determined by ELISA, GR and p65 protein was assessed by Western Blot, mRNA by RT-qPCR and p65 recruitment to the CCL11 promoter by ChIP assay.

## Results

CCL11 release was higher in ASMC of non-severe but not severe asthmatics and non-asthmatic controls; CXCL8 release was similar in all groups. In severe asthmatics dex (10^-6^ M) caused less suppression of CCL11 (42% vs 14%, p<0.05) and CXCL8 (47.88% vs 26.7%, p<0.05) compared to non-severe asthmatics or healthy controls, respectively. TNFα-induced phospho-p38 mitogen-activated protein kinase was increased in severe asthmatic ASMC compared to non-severe and non-asthmatics while a p38 inhibitor increased the inhibitory effect of dex. GR expressed in severe and non-severe asthma was 49% of that in the healthy (p<0.01). Dexamethasone-induced GR nuclear translocation at 2h, in SA, was 60% of that in either the healthy or NSA (p<0.05). TNF-α induced greater p65 mRNA expression in SA whereas baseline and TNFα-induced nuclear abundance, and dexamethasone suppression of p65 expression, were similar between groups. While not modulating p65 nuclear translocation, dex attenuated p65 recruitment to the CCL11 promoter in the healthy and NSA, but this suppressive effect was impaired in SA.

## Conclusions

Airway smooth muscle cells of patients with severe asthma are corticosteroid insensitive; this may be secondary to heightened p38 mitogen-activated protein kinase. Decreased GR expression with impaired nuclear translocation in ASMC, associated with reduced dexamethasone-mediated attenuation of p65 recruitment to gene promoters, may underlie the mechanism of CS insensitivity in severe asthma.

